# Evidence of parental care as a newly identified reproductive isolating barrier

**DOI:** 10.1111/1365-2656.70348

**Published:** 2026-09-23

**Authors:** Colby Behrens, Meghan F. Maciejewski, Alexandra Sumarli, Gina Lucas, German Lagunas‐Robles, Kieran Samuk, Alison M. Bell

**Affiliations:** ^1^ Department of Evolution, Ecology and Behavior, School of Integrative Biology University of Illinois Urbana‐Champaign Urbana Illinois USA; ^2^ Department of Evolution, Ecology, and Organismal Biology University of California, Riverside Riverside California USA; ^3^ Department of Biology Indiana University Bloomington Indiana USA; ^4^ Program in Ecology, Evolution and Conservation University of Illinois Urbana‐Champaign Urbana Illinois USA; ^5^ Carl R. Woese Institute for Genomic Biology University of Illinois Urbana‐Champaign Urbana Illinois USA; ^6^ Program in Neuroscience University of Illinois Urbana‐Champaign Urbana Illinois USA

**Keywords:** filial cannibalism, hybridization, parental care, reproductive isolation, speciation, stickleback

## Abstract

1. Variation in behaviour can contribute to reproductive isolation by preventing gene flow among populations.
2. Here, we tested the novel hypothesis that parental care, when dysregulated, can function as a reproductive isolating mechanism in three‐spined stickleback fish (*Gasterosteus aculeatus*). In the typical ‘common’ stickleback ecotype, males provide care to their offspring by fanning with their pectoral fins and defending their nest. By contrast, a divergent ‘white’ stickleback ecotype has evolutionarily lost care and disperses embryos into the surrounding environment.
3. We examined how paternal care from common, white and F1 hybrid fathers influenced offspring survival. We detected no intrinsic incompatibilities in embryos, but F1 hybrid fathers exhibited dysregulated parental care that coincided with decreased survival of parented offspring. The increased offspring mortality may be explained by further dysregulation of feeding and parenting circuitry, as F2 hybrids exhibited significantly higher rates of post‐fertilization filial cannibalism than common or white fathers.
4. Additionally, despite strong divergence in nesting–building and courtship behaviour, F1 hybrids achieved mating success at a similar rate as male commons and whites, suggesting that prezygotic barriers against hybrids may be weak.
5. The observed postzygotic isolation, and potentially weak prezygotic isolation in lab‐based studies, suggest that hybridization is likely occurring at low rates in the wild. Population genetic analysis supported this, as low proportions of putative hybrids were detected in sympatric sites.
6. Together, these results may help explain why genetic divergence between these phenotypically distinct ecotypes is low and provide evidence that dysregulated parental behaviour can act as a newly discovered postzygotic reproductive isolating barrier.

## Graphical abstract

Behavioural variation can contribute to reproductive isolation and can be an important driver of speciation. Here, we show that a previously undescribed mechanism, parental care, can function as a postzygotic reproductive isolating barrier when dysregulated in hybrids.

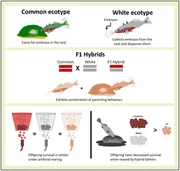

## 1 INTRODUCTION

Behaviour can directly affect survival and reproductive success and therefore plays an important role in evolutionary processes, including the evolution of reproductive isolating barriers (Coyne & Orr, 2004). For example, behaviours like courtship displays, territoriality and song can facilitate speciation if they are involved in preventing the formation of zygotes between different populations, thereby acting as prezygotic reproductive isolating barriers (Coyne & Orr, 1989; Fitzpatrick & Gray, 2001; Lackey & Boughman, 2017; Silberglied & Taylor, 1978). Behaviour can also act as a postzygotic isolating barrier if, for example hybrids display altered behaviours that are important for survival or reproductive success, such as intermediate migration routes (Delmore & Irwin, 2014; Helbig, 1991), hampered cognition (McQuillan et al., 2018) or dysregulated courtship behaviour that falls outside of the normal adaptive range (Clark et al., 2010; Gottsberger & Mayer, 2007; Noor, 1997; Pashley & Martin, 1987).

Here, we investigate dysregulated parental care as a previously undescribed behavioural postzygotic isolating barrier in animals. Although parental care is relatively rare in nature, it has evolved independently multiple times and is critical for the development and survival of offspring in those groups (Clutton‐Brock, 1991). Successful rearing often requires proper expression of a suite of behaviours, including resource allocation, brood hygiene and predator defence, and disruption of any of these could reduce offspring survival. Importantly, rearing failure could also result from the exaggeration of behaviours. For example, though filial cannibalism is relatively common in nature (Klug & Bonsall, 2007; Polis, 1981), a moderate increase in this behaviour could significantly increase mortality in a clutch. Therefore, hybrids that exhibit altered or dysregulated parental behaviour in any number of these traits may have low fitness, even if no gametic or other intrinsic incompatibilities are present.

In this study, we test the hypothesis that dysregulated parental behaviour can act as a postzygotic reproductive isolating barrier in three‐spined stickleback fish (*Gasterosteus aculeatus*), a classic model for speciation (McKinnon & Rundle, 2002). Stickleback are famous for their propensity to adapt to new environments, and the repeated colonization of freshwater habitats by marine ancestors has resulted in the radiation of sticklebacks throughout the Northern hemisphere. Studies of ‘species pairs’ of phenotypically divergent populations, for example benthic‐limnetic (Arnegard et al., 2014; Bay et al., 2017; Keagy et al., 2016; McPhail, 1992), lake‐stream (Hendry et al., 2002; Räsänen & Hendry, 2014), freshwater‐anadromous (Aguirre et al., 2022; Hay & McPhail, 1975; Ziuganov, 1995), resident‐anadromous (Dean et al., 2019) and Japan Sea stickleback (Kitano et al., 2007, 2009) have offered strong support for the importance of ecological drivers of divergence in sticklebacks.

A less‐studied pair of populations are the ‘white’ and ‘common’ stickleback ecotypes found in Nova Scotia, Canada. Whites and commons occupy the same trophic niche (Samuk, 2016), yet they exhibit markedly divergent reproductive behaviour, especially parental care. As is typical of the family Gasterosteidae, males of the common stickleback ecotype provide parental care for several days by aerating their embryos via pectoral fanning and by removing decaying eggs, which is critical to preventing fungal infections (Wootton, 1976). By contrast, the divergent white ecotype has evolutionarily lost care. After fertilizing the eggs, white stickleback fathers remove embryos from the nest and disperse them into the surrounding environment (Behrens et al., 2024; Blouw, 1996; Blouw & Hagen, 1990). Commons and whites differ notably in body size (Blouw & Hagen, 1990), and males differ in several other traits important for reproduction including nest architecture (male whites build relatively loose nests) and courtship behaviour (male whites engage in high rates of female‐directed courtship behaviour (Behrens et al., 2024; Haley et al., 2019; Jamieson et al., 1992)). Female whites and commons have also diverged in reproductive traits, with female whites producing relatively large clutches of small eggs (Behrens et al., 2025; Grant, 1993). Female whites produce clutches of eggs that do not stick together (Grant, 1993), which may facilitate the embryo scattering behaviour of male whites and prevent the spread of fungus throughout the entire clutch.

Population genetic analyses suggest that the ecotypes diverged recently (<12 K years) and are genetically distinct, but their genome‐wide differentiation is low (*F*_ST_ ~ 0.02) and there is likely gene flow between them (Samuk, 2016; Sumarli, A. & Samuk, K. personal communication). The populations frequently occur in sympatry, but there are currently no known sites where only whites are found. There is no evidence of altered embryonic development or inviable gametes in F1 hybrids or their offspring: laboratory‐generated F1 and F2 hybrids are fertile and produce viable offspring (Behrens et al., 2024, 2025; Blouw, 1996), suggesting that hybridization could be occurring in the wild. It is possible that partially non‐overlapping breeding seasons and/or assortative mating contribute to prezygotic isolation in this system, but evidence is mixed (Blouw & Hagen, 1990; Corney & Weir, 2023). Altogether, it is unclear what processes generated and maintained such extensive phenotypic divergence without a similar genetic divergence, and the rate at which hybrids occur in natural populations is unknown.

Here, we test the novel hypothesis that dysregulated parental behaviour can be a form of behavioural sterility (Coyne & Orr, 2004) and act as an extrinsic postzygotic reproductive isolating barrier by preventing F1 hybrids from achieving reproductive success. In the white‐common stickleback system, this predicts that even if the white and common ecotypes hybridize in the wild, the two ecotypes fail to mix completely because F1 hybrid males exhibit dysregulated paternal behaviour that results in low offspring survival. This hypothesis is motivated by a previous study showing that F1 hybrid white × common males perform a mixture of typical white and common behaviours after mating, with relatively low levels of the behaviours characterizing each strategy (i.e. low levels of dispersal behaviour and low levels of fanning (Behrens et al., 2024; Blouw, 1996)). Given the importance of paternal care for offspring survival in this species (Wootton, 1976), we predicted that dysregulated behaviour in F1 hybrid fathers would result in low offspring survival.

To test this hypothesis, we quantified multiple potential mechanisms of reproductive isolation in this system. First, we compared the survival of embryos reared by white or common fathers to the survival of embryos that received care from F1 hybrid fathers. If dysregulated paternal behaviour acts as a postzygotic reproductive isolating barrier in this system, then we predicted that embryos that received care from F1 hybrid fathers would have lower survival than embryos that received care from white or common fathers. In addition to measuring offspring survival, we also measured paternal behaviour to gain insights into the behavioural mechanisms that may contribute to behavioural sterility, including behaviours that reduce care or increase offspring mortality. To control for potential intrinsic barriers, we measured the survival of offspring that were reared artificially, which generates high rates of embryo survival if there are no genetic incompatibilities or gametic sterility (Day et al., 1994), and compared the survival of artificially‐reared offspring sired by F1 hybrid fathers to the survival of offspring sired by white or common males.

Like many fishes, male stickleback often consume eggs from conspecifics' nests (FitzGerald, 1991) as well as their own (filial cannibalism). The causes of filial cannibalism in sticklebacks are not well understood (see Mehlis et al., 2009), but we suspected that if paternal care was dysregulated in hybrid fathers, then it could manifest as higher than usual rates of filial cannibalism, as feeding and parenting neural circuits are closely linked in vertebrate brains (Fischer & O'Connell, 2017). If hybrid males possess dysregulated parental circuits, this dysregulation could also elevate feeding‐related behaviours like filial cannibalism. We therefore examined rates of filial cannibalism by recently mated male ‘commons’, ‘whites’ and hybrids, and predicted that hybrids would exhibit higher rates of cannibalism than either parental ecotype.

For completeness, we also explored the possibility of mate choice acting as a prezygotic barrier against hybrid males. White and common males employ unique courtship and nesting strategies throughout the reproduction season (Behrens et al., 2024), and the intermediate displays of hybrid males could appear unattractive to potential female mates. We therefore examined the mating success of hybrid males and investigated the potential role of nest architecture as an isolating barrier preventing the formation of backcross hybrids.

Finally, we examine the prevalence of hybrids across natural populations of white and common stickleback. Though gene flow is likely ongoing in this system (Samuk, 2016), the presence and rate of hybridization have never been examined in wild populations and could inform the interpretation and implications of this lab‐based study for isolating barriers in natural populations. Here, we bring evidence to bear from a population genomic survey which shows that hybridization occurs in the wild.

## 2 METHODS

### 2.1 Animal collection and husbandry

In 2023, three‐spined stickleback were collected from two sites in Nova Scotia, Canada. White sticklebacks were collected at Salmon River (45°21′9.57″ N 61°28′22.20″ W) and common sticklebacks were collected from Blue's Cove (45°53′55.7″ N 61°05′11.1″ W) using unbaited minnow traps. Because white stickleback are absent from Blue's Cove, all males collected at that site were unambiguously classified as common stickleback. Both ecotypes occur at Salmon River, so we differentiated whites from commons using known differences in body size, nuptial coloration and egg stickiness (Behrens et al., 2025; Blouw & Hagen, 1990). Hybrid stickleback are typically phenotypically intermediate (Behrens et al., 2024), and no morphologically intermediate individuals were collected at either site. Clutches of white, common and a full reciprocal cross of F1 hybrids were then generated through artificial fertilization, where testes from sacrificed males were macerated and applied over clutches of eggs squeezed from females. Embryos were reared at the University of California Riverside (Methods S1) and later transported to the University of Illinois Urbana‐Champaign for experimental testing.

### 2.2 Sample collection for population genetic analyses

In June 2019, adult common stickleback were collected from Cherry Burton Road (N 46° 01.516′ W 64° 06.150′) and white stickleback were collected from Canal Lake (44°29′54.0″ N 63°54′09.1″ W) and transported to the University of Illinois in Urbana‐Champaign before tissue collection. In the spring of 2022, random fin clips were obtained from 50 additional three‐spined sticklebacks from each of four sites in Nova Scotia, two where white and commons are known to be sympatric (Canal Lake and Salmon River, coordinates above) and two where only commons are found (Blues Cove, discussed previous, and McIvor Road Junction, 45°56′09″ N 61°04′06″ W; Methods S2). There are no known sites where only whites are found.

### 2.3 Behavioural phenotyping and offspring survival

Upon entering breeding condition, signified by red throats and blue eyes, 1‐year‐old male commons, whites, W♀ × C♂ F1s and C♀ × W♂ F1s were housed in individual tanks and provided with nesting materials (Behrens et al., 2024). Male commons and whites were paired with gravid females from their respective populations, and males of both F1 hybrid cross types were mated to female commons or female whites, resulting in males in 6 different conditions, with *n* = 6 males per condition. Whites and commons (F0s) are expected to be more abundant than F1 hybrids in the wild, so we did not test intercross (F1 hybrid × F1 hybrid) conditions. We quantified the architecture of male nests if they were visible without manipulation. Briefly, nests received qualitative scores on four attributes (height, opening, sand content, location) that were combined in a single nesting metric (Behrens et al., 2024). We then introduced a live gravid female into the male's tank to spawn via no‐choice trials (Boughman, 2001). The female was weighed, placed into a male tank and removed if a mating did not occur within 15 min. If a mating did occur, the female was removed and reweighed to estimate the mass of the clutch. We tracked the number of females that were introduced to each male before successfully mating as a rough proxy for mate preference. After fertilization, male parenting behaviours, including fanning and embryo dispersal (Behrens et al., 2024; Blouw, 1996; Wootton, 1976), were recorded twice daily for 15 min for 4 days with the program JWatcher (Blumstein et al., 2006) in order to gain insights into the behavioural determinants of successful reproduction.

Fathers were removed from their tanks 4 days after fertilization, which is when many parenting behaviours begin to peak (Behrens et al., 2024). This timepoint was chosen to accurately quantify embryo survival during peak parental care; therefore, differences in survival may be conservative estimates relative to effects observed in later stages of care. Males were euthanized and testes were removed to artificially fertilize an additional clutch of eggs that was artificially reared (Day et al., 1994); clutch size of artificially‐reared clutches was manually counted immediately after fertilization. The same female ecotype was used for artificially‐ and father‐reared clutches for each male.

Embryos from natural fertilizations were also collected at this time to estimate the survival of father‐reared offspring. To collect embryos located in the nest, we placed the nest under a dissection microscope and sifted through algal strands with fine forceps. Care was taken to ensure that embryos were not dropped into the tank during nest removal. White and hybrid stickleback disperse embryos into the surrounding environment; therefore, a reliable estimate of offspring survival requires a survey of the entire tank. However, stickleback embryos are transparent and difficult to identify while resting on substrate, especially during early stages of development (Swarup, 1958). To account for this, we removed tank substrate (e.g. gravel) in increments of <240 mL, spread the substrate loosely across small tanks (dimensions 7 × 4 × 5”) and used plastic dropper pipettes to push any embryos into the water column for detection and counting. Tanks from all males, including the non‐dispersing commons, were surveyed. Healthy embryos are readily identifiable by eye capsules and blood circulation at this stage in development (Swarup, 1958) so only living embryos were collected and saved. This method enabled estimation of embryo survival regardless of parenting strategy with high confidence. All embryos were then counted and artificially incubated until hatching.

### 2.4 Filial cannibalism

To quantify rates of cannibalism, the stomachs of males were dissected and the number of eggs/embryos was counted within 1 h after mating, when the critical decision about whether to disperse (whites) or care for embryos (commons) is made. Data on filial cannibalism by the common, white or F1 hybrid males in the survival study were not available because their parenting behaviour was observed for 4 days after mating, and rates of cannibalism are low at that point. A separate set of whites and commons (*n* = 22 per ecotype) was assessed for filial cannibalism across 2021 and 2022, and filial cannibalism of F2 hybrids (originating from a single white ♀ × common ♂ cross, *n* = 77) was quantified in 2021 (Behrens, Tucker, et al., 2026a). We therefore test the hypothesis that hybridization influences filial cannibalism using filial cannibalism rates of F2 hybrids as an indirect proxy. Male whites and commons were paired with females from their own ecotypes, and F2 hybrids were paired with female whites. Males from 2021 (common, white and F2 hybrid) had body mass recorded immediately prior to dissection. Males were consistently fed in the morning prior to mating to prevent differences in satiation from affecting rates of cannibalism. The fertilization rate of cannibalized eggs was recorded to assess whether filial cannibalism was preferentially directed toward unfertilized eggs, which are susceptible to fungal infections that could threaten the entire clutch (Wootton, 1976).

### 2.5 Population genetics

#### 2.5.1 DNA extractions and library prep

We performed standard phenol‐chloroform DNA extractions (Sambrook et al., 1989) to extract DNA from the 200 randomly sampled individuals from 2022 (collected from Canal Lake, Salmon River, Blues Cove and McIvor Road Junction) as well as 20 common sticklebacks (10 male, 10 female) from Cherry Burton Road and 20 white sticklebacks (10 male, 10 female) from Canal Lake collected in 2019.

DNA from the 200 randomly sampled individuals was normalized and prepared for double digest restriction‐site associated DNA (ddRAD) sequencing using published methods (Brelsford et al., 2016), which implements elements proposed by Parchman et al., 2012 and Peterson et al., 2012, with the restriction enzymes PstI and MseI and removed small DNA fragments with Omega magnetic beads (OMEGA Bio‐Tek). The remaining 40 samples were sent to the UC Davis DNA Technologies and Expression Analysis Core Facility for Whole Genome Sequencing (WGS) library prep. All sequencing (RAD and WGS libraries) was performed at the UC Davis DNA Technologies and Expression Analysis Core Facility on an Illumina High Seq‐X. Variants were then called on RADseq and WGS reads (Methods S3).

#### 2.5.2 Principal component and hybrid analysis

The extremely low genetic differentiation between white and common sticklebacks precludes standard analyses aimed at hybrid classification (e.g. triangle plots). To identify potential hybrids, we performed principal component analysis (PCA) on the jointly filtered SNPs from the RADseq and WGS data. We used PCA instead of other commonly used model‐based methods (e.g. ADMIXTURE) because it does not make any assumptions about a fixed number of discrete populations in our data. We first performed PCA on the total dataset. Because sex and ecotype identity were unknown in the randomly sampled fish, we assigned sex and ecotype genotypically. Sex was assigned by identifying individuals with high numbers of homozygous Y‐linked SNPs in the non‐PAR region of the Y‐chromosome. Ecotype identity was assigned by examining the co‐clustering of RADseq samples with WGS samples (which have known ecotype identity). Following this procedure, we then examined patterns of clustering in the randomly sampled populations and identified intermediate individuals falling between two genotypic clusters. To further test for the presence of hybrid individuals, we performed two additional ancestry analyses on the sympatric and contact‐zone samples (Cherry Burton Road *n* = 20 WGS, Canal Lake *n* = 70 [20 WGS + 50 RAD], Salmon River *n* = 39 RAD; 129 individuals total). The allopatric Blues Cove and McIvor Road Junction populations, which form a single tight cluster in the PCA, were excluded from both analyses.

We first performed a supervised Discriminant Analysis of Principal Components (DAPC; Jombart et al., 2010) using the *adegenet* R package. RAD ecotype labels in our sample sheet were originally assigned post‐hoc by PCA, so training a DAPC on those labels would simply rediscover the PCA boundary. To avoid this circularity, we trained the DAPC on the 40 WGS samples only, using their a priori morphological labels (Cherry Burton common, Canal Lake white), and then predicted the ecotype of the 89 RAD‐sequenced individuals as held‐out via predict.dapc(). The number of retained principal components was chosen by cross‐validation within the WGS training set (xvalDapc, 100 replicates, 90/10 split, n.pca.max = 30); n.da was fixed at 1 (two‐group fit). Mean imputation of missing genotypes pulls held‐out RAD samples toward the discriminant midpoint, producing an apparent excess of intermediate individuals. To minimize this artefact, SNPs were restricted to those with ≤5% missingness in the RAD subset prior to imputation (2318 SNPs after filtering).

We then performed sparse non‐negative matrix factorization (sNMF; Frichot et al., 2014) using the *LEA* R package. sNMF estimates individual ancestry proportions without prior labels and is therefore an unsupervised complement to the supervised DAPC. Per‐SNP missingness and minor allele frequency were recomputed on the 129‐sample mainland subset (biallelic, MAF ≥0.03, depth/GQ/missingness/allele‐balance filters as above), and no LD pruning was applied. We ran snmf() for *K* = 1–6 with 100 replicates per *K* and chose the best run per *K* by minimum cross‐entropy.

### 2.6 Statistical analysis

We compared the survival of father‐reared offspring of F1 hybrid males (W♀ × C♂ and C♀ × W♂) to the survival of father‐reared offspring of F0 fathers (common and white). Cross types were lumped to improve statistical power in survival analyses. The survival of father‐reared offspring was estimated by physically counting the number of embryos remaining in the tank 4 days after fertilization, as described above. To confirm that intrinsic hybrid incompatibilities and/or sterility do not contribute to reproductive isolation in this system, we also compared the survival of artificially‐reared embryos sired by F0 versus F1 hybrid males. The survival of artificially‐reared offspring was quantified by counting the number of surviving embryos after 4 days of artificial incubation. Differences in clutch size were accounted for statistically by dividing the number of surviving embryos by the original clutch mass in father‐reared clutches and by dividing the number of surviving embryos by the original number of eggs in the clutch in artificially‐reared clutches. Father‐reared offspring survival (surviving embryo count per mg clutch mass) was normally distributed and was analysed via a linear model. Survival of artificially‐reared offspring (surviving embryos per total eggs) was highly right‐skewed and fit a beta distribution according to goodness‐of‐fit statistics. We therefore analysed artificially‐reared survival as a proportion in a Beta regression model using the *betareg* function of the package *betareg*. A small number (0.001) was added or subtracted to the proportion if necessary to ensure values were between 0 and 1. The paternal cross type (F0, F1), maternal cross type (common, white) and their interaction were included as fixed effects in both models. In both cases, we compared full and reduced models with AIC and likelihood ratio tests and retained models with the best fit.

In the filial cannibalism experiment, the pre‐fertilization mass of males was recorded but the mass and egg count of female clutches were unavailable. Filial cannibalism was therefore quantified using raw counts of eggs found in the stomach of a male. This dataset was non‐normal and analysed with nonparametric statistical tests.

Courtship success (number of trials before successfully mating) data were discrete and right‐skewed, so differences among male cross types were tested via nonparametric Kruskal–Wallis tests. Separate tests were performed for each female cross type. Nest architecture scores were non‐normal and analysed with nonparametric tests (Kruskal–Wallis and Spearman rank correlations). Parenting behaviours of stickleback are known to differ across time and populations (Behrens et al., 2024), so we examined two timepoints that are known to differ across groups: immediately after fertilization (day 0) and 4 days after fertilization (day 4). Parenting behaviours were fitted to linear models with the cross types of the male, female and their interaction as fixed effects. Dispersal data were overdispersed and zero‐inflated but approximated a normal distribution after log+1 transformation. AIC‐based model comparison indicated a greater fit with transformed values in a linear model than raw values in a negative binomial model (ΔAIC = 92). Similarly, fanning data were not normally distributed, but square‐root transformation yielded significantly stronger support than a Gamma GLM (ΔAIC = 163). Models of transformed data were therefore retained for both behaviours. Full and additive models were also compared for both behaviours. Interaction terms were not supported for dispersal (ΔAIC = 1.994) or day 0 fanning (ΔAIC = 1.744) behaviours, so additive models were retained. However, the interaction term improved the fit for day 4 fanning behaviours (ΔAIC = 1.019), so the full model was retained.

Analyses were performed in R version 4.2.1 (R Development Core, 2011) and visualized with the package ‘ggplot2’ (Wickham, 2011).

## 3 RESULTS

### 3.1 Offspring of F1 hybrid males had low survival when reared by their father

To test the hypothesis that offspring reared by F1 hybrid males suffers greater mortality than offspring reared by F0 males, we compared the survival of father‐reared offspring of F1 hybrid males (W♀ × C♂ and C♀ × W♂) to the survival of father‐reared offspring that were reared by F0 fathers (common and white). The interaction term was not supported (ΔAIC = 1.999), so the additive model was retained. Paternal cross type had a significant effect on survival in father‐reared clutches (Figure 1d; male *F*_1,33_ = 4.32, *p* = 0.046; female *F*_1,33_ = 1.91, *p* = 0.176), with offspring of F0 fathers exhibiting higher survival than offspring of F1 hybrid fathers.

**FIGURE 1:**
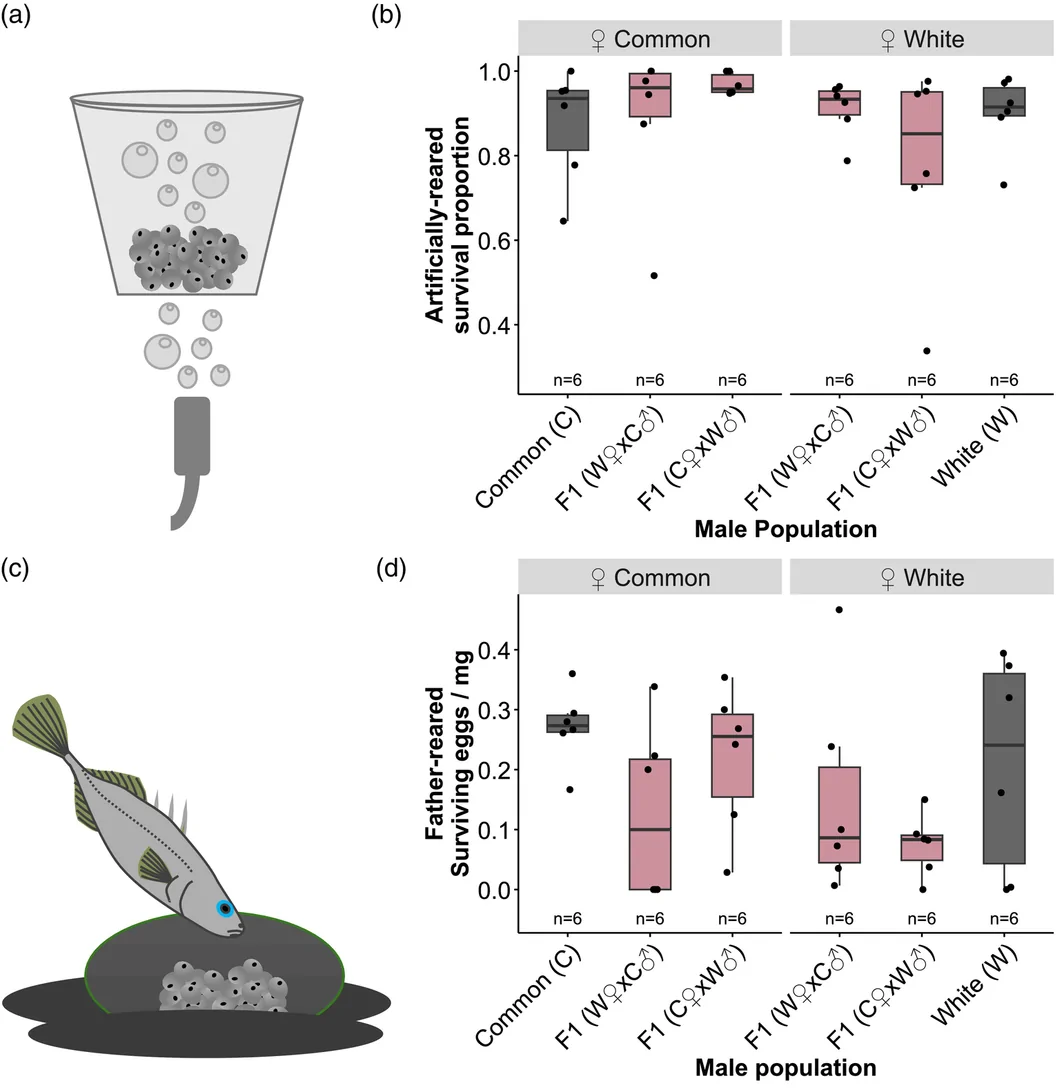
Survival of offspring under artificial and father‐rearing. (a) Embryos undergo artificial‐rearing over a bubbling airstone, and (b) offspring do not differ in survival under artificial‐rearing conditions. (c) Father‐reared offspring remain in the nest, and (d) model testing suggests that offspring from F0 fathers exhibited significantly higher survival under father‐rearing conditions. The paternal population is shown on the *x*‐axis and plots are faceted by the maternal population. Boxes are coloured by paternal population (F0 in grey, F1 in pink). Each data point represents a clutch, with boxes representing interquartile ranges with the median and whiskers representing 1.5 times interquartile ranges. Post‐hoc tests did not detect significant differences among groups.

To confirm that intrinsic hybrid incompatibilities do not contribute to reproductive isolation in this system, we compared the survival of artificially‐reared embryos that were sired by F0 versus F1 hybrid males. The interaction term was not supported (ΔAIC = 0.753), so the additive model was retained. The survival of artificially‐reared embryos differed according to the cross type of the mother but not the father (Figure 1b; male *χ* ^2^ = 0.09, *p* = 0.764; female *χ* ^2^ = 4.29, *p* = 0.038), suggesting a lack of intrinsic hybrid incompatibilities in this system.

Common and white clutches did not significantly differ in mass in artificially‐reared (Figure S1; *t* = 0.27, df = 32.1, *p*‐value = 0.79) or father‐reared (Figure S1; *t*_31.9_ = −0.71, *p* = 0.49) clutches, consistent with previous studies (Behrens et al., 2025).

### 3.2 Dysregulated paternal behaviour of F1 hybrid males contributed to low rates of offspring survival

Because offspring reared by F1 hybrid males had lower survival, we next examined the parenting behaviours that may have contributed to this lower survivorship. In general, the parenting behaviour of F1 hybrid males was intermediate between whites and commons, and F1 hybrid males exhibited all combinations of elements of the white and common strategies (Figure 2). For example, some F1 hybrid individuals exhibited relatively high dispersal and fanning rates, while other hybrid individuals exhibited low levels of both behaviours.

**FIGURE 2:**
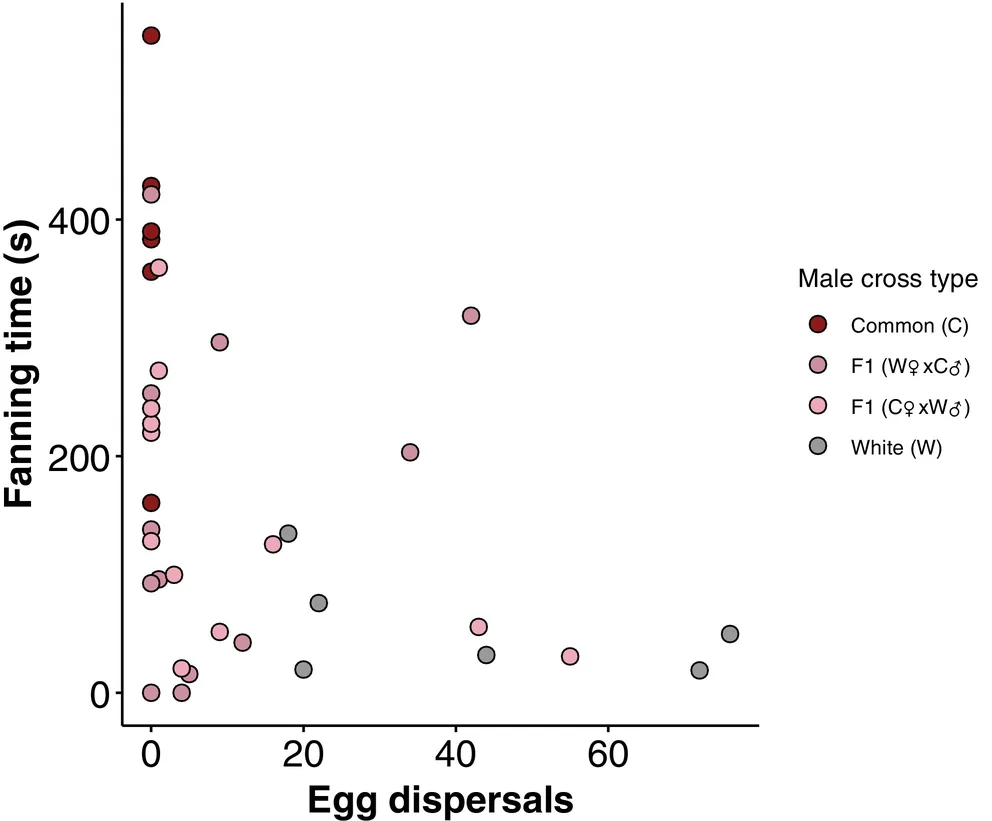
Relationship between the dispersal rate after fertilization and nest fanning four days after fertilization. Data points are coloured by cross type. F1 hybrids exhibit a mix of typical white and common behaviours.

Male commons never dispersed embryos (Figure 3A; Video S1), consistent with previous studies (Behrens et al., 2024; Blouw, 1996). By contrast, male whites and F1 hybrids dispersed embryos from their nest (Video S2; Video S3), but the number of dispersals depended on the cross type of both the male and female (Figure 3A; male *F*_3,31_ = 15.25, *p* < 0.001; female *F*_1,31_ = 25.20, *p* < 0.001), with embryos from female whites being more readily dispersed than embryos from female commons.

**FIGURE 3:**
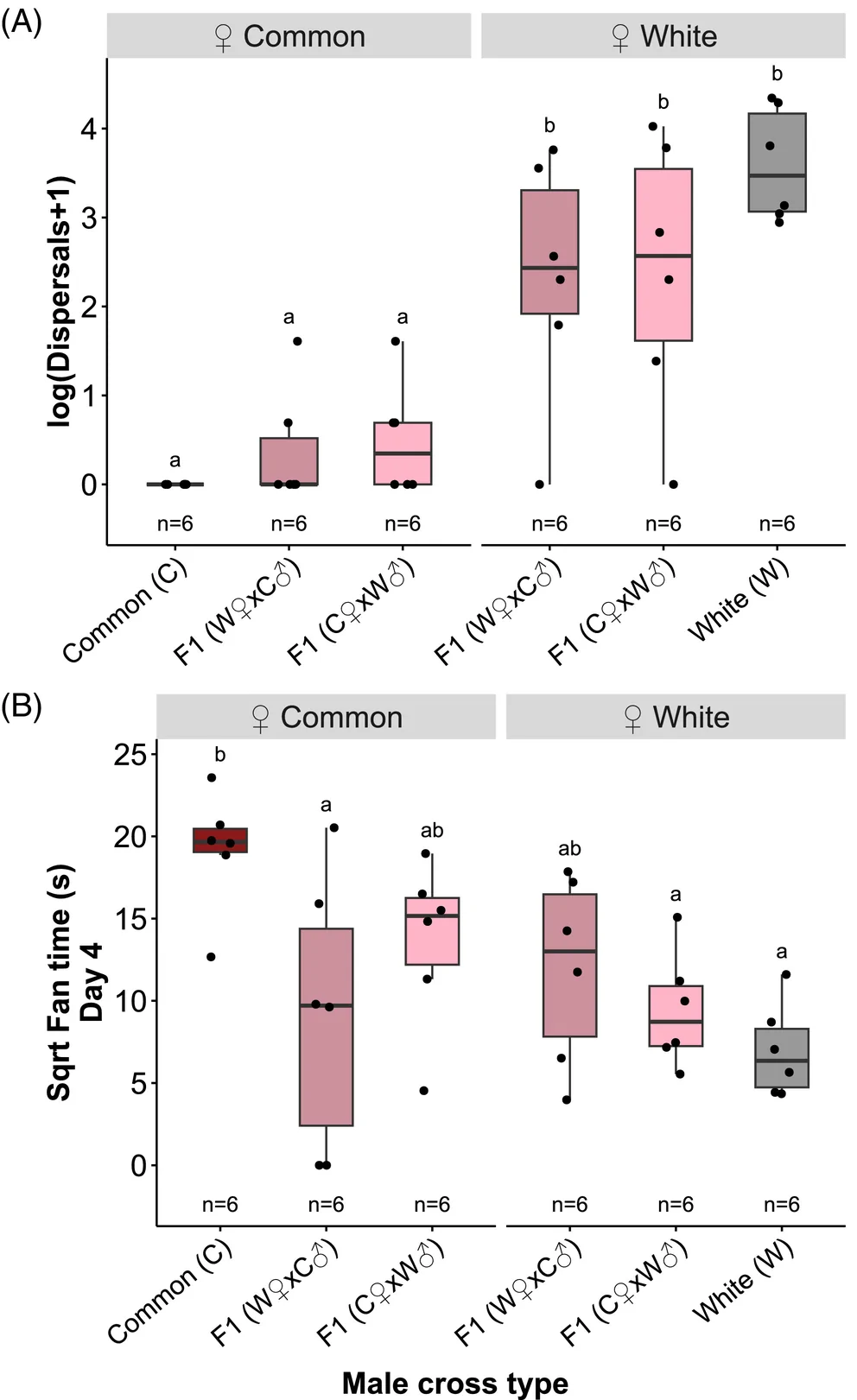
Differences in paternal care across male and female cross types. (A) Fathers dispersed eggs from white clutches more frequently than eggs from common clutches. (B) Male commons fanned their eggs more than male whites, and F1 hybrids were often intermediate. Plots are faceted by female cross type. Each data point represents a male, with boxes representing interquartile ranges with the median and whiskers representing 1.5 times interquartile ranges. Significance lettering reflects Tukey HSD post‐hoc tests.

As embryos develop, stickleback fathers typically increase their fanning rate to provide aeration. We did not detect differences in fanning immediately after fertilization (male *F*_3,31_ = 1.51, *p* = 0.232; female *F*_1,31_ = 0.89, *p* = 0.353) but male commons exhibited significantly higher rates of fanning than male whites and F1 hybrids at 4 days after fertilization (Figure 3B; male *F*_3,30_ = 6.09, *p* = 0.002; female *F*_1,30_ = 0.14, *p* = 0.71; male: female *F*_1,30_ = 2.62, *p* = 0.116). Whether the male was mated to a white or common female did not influence levels of fanning behaviour in F1 hybrids (Figure 3B).

One possible explanation for the behavioural sterility of F1 hybrid males is that they failed to modulate their behaviour in response to cues from embryos by, for example failing to fan their embryos, which caused the embryos to die. Indeed, clutches from F1 hybrids that received more fanning had higher rates of survival (dispersal F_1,20_ = 2.93, *p* = 0.102; fanning *F*_1,20_ = 11.29, *p* = 0.003; dispersal: fanning *F*_1,20_ = 4.60, *p* = 0.044). However, contrary to this hypothesis, fanning was significantly associated with the number of embryos in a nest (Figure 4; *r* = 0.68, *p* < 0.001), and both F1 hybrid crosses appeared to modulate their fanning rates according to the number of remaining embryos in their nests (F1 W♀ × C♂ *r* = 0.55, *p* = 0.062; F1 C♀ × W♂ *r* = 0.70, *p* = 0.011). Together, these results suggest that F1 hybrid males are capable of providing care; they attended to cues of embryos in their nest and modulated their behaviour accordingly, but their offspring did not survive for other reasons.

**FIGURE 4:**
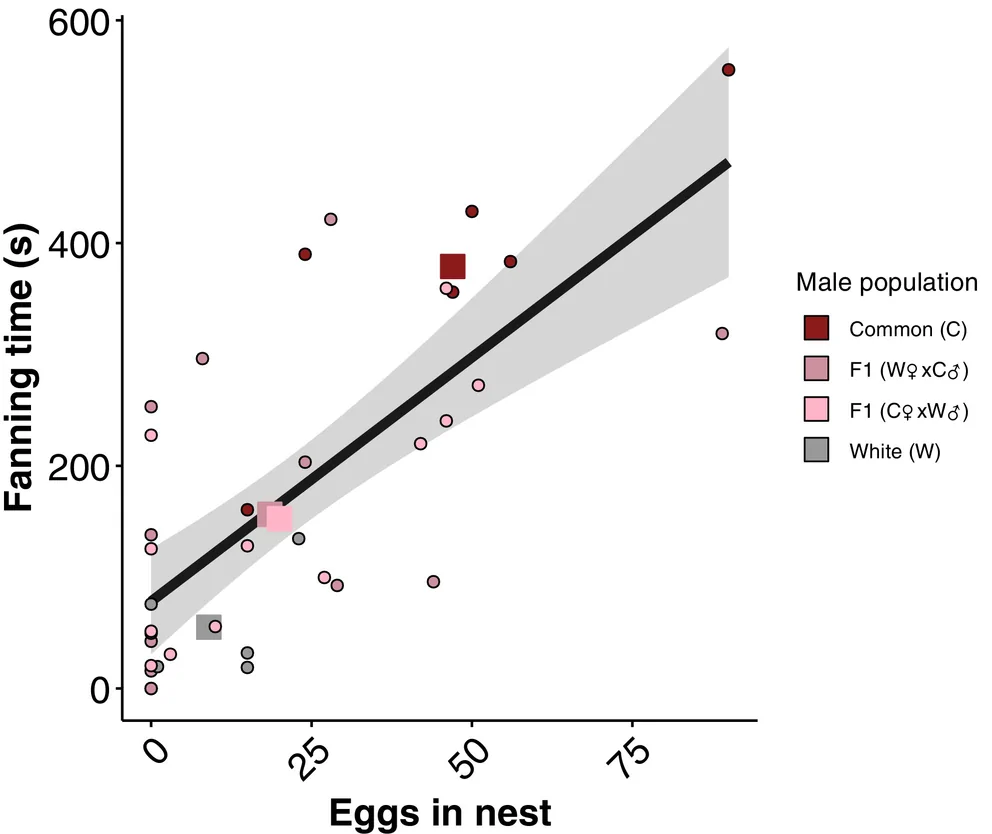
Relationship between fanning rate and the surviving embryos in a nest 4 days after fertilization. Data points are coloured by cross type. Small circles represent individuals and large squares represent group means. The regression line is shown with its 95% confidence interval. Stickleback fathers fan more when more embryos are present in the nest.

Therefore, we investigated another behavioural mechanism that may have contributed to the low survival of offspring of F1 hybrid father‐reared offspring: filial cannibalism. Consistent with the hypothesis that hybrid fathers have poor reproductive success due to filial cannibalism, F2 hybrid males had many more eggs/embryos in their stomachs (*μ* = 26.5, SD 18.3, *n* = 77) relative to either white (*μ* = 6.77, SD 6.55, *n* = 22) or common (*μ* = 2.14, SD 3.04, *n* = 22) males (Figure 5; Kruskal–Wallis, *χ* ^2^ = 58.33, df = 2, *p* < 0.001). We found no evidence that F2 hybrids engaged in high rates of filial cannibalism because they were in poor condition or small: Both male commons and F2 hybrids were heavier than male whites (Kruskal–Wallis, *χ* ^2^ = 6.40, df = 2, *p* < 0.041), and F2 hybrids consumed significantly more eggs than commons or whites even when corrected for body mass (Kruskal–Wallis, *χ* ^2^ = 30.85, df = 2, *p* < 0.001). We detected a large proportion of fertilized embryos in F2 males' stomachs (36% of eggs were unbroken, 67% of unbroken eggs were fertilized). Although the fertilization status of the majority of eggs was unclear due to mastication, the results suggest that hybrid males may not preferentially consume unfertilized eggs that could threaten the entire clutch. Instead, they appear to have consumed eggs regardless of viability.

**FIGURE 5:**
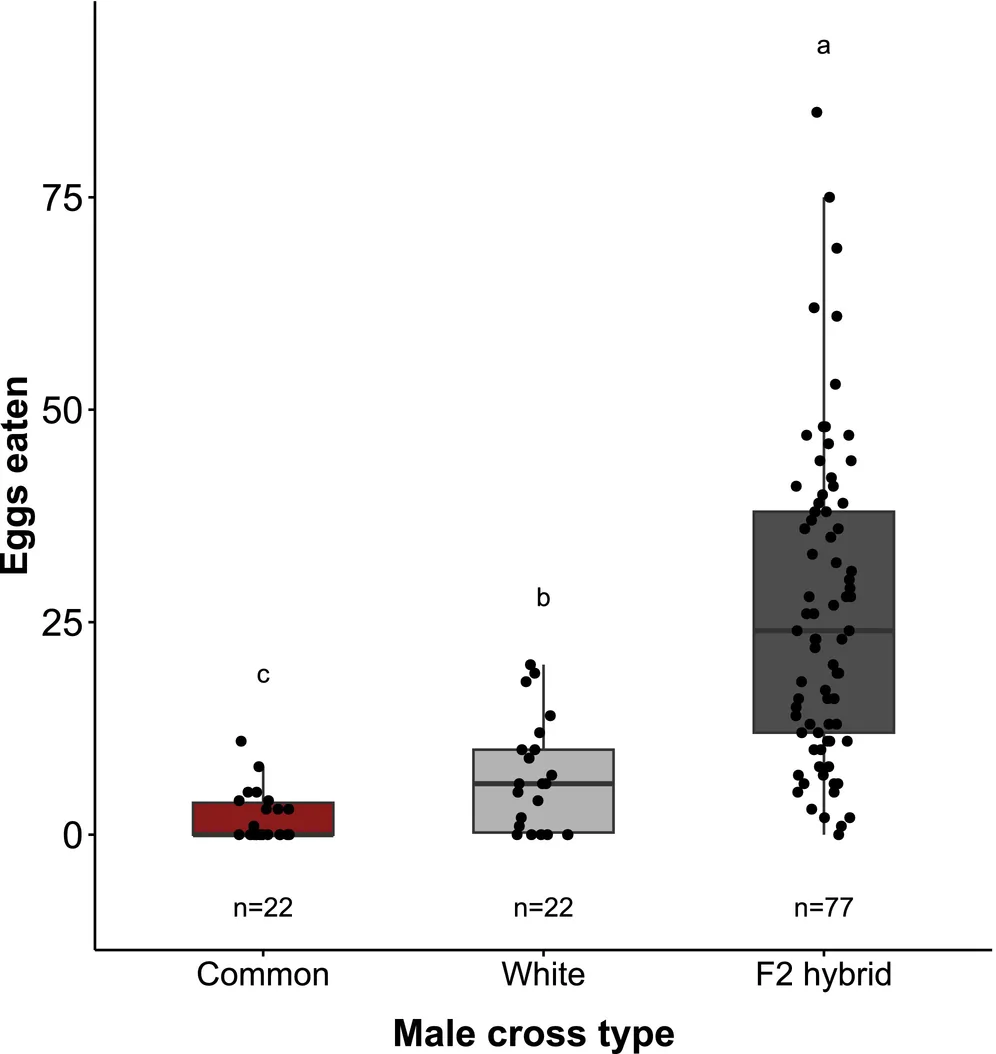
Cannibalism rates in stickleback fathers. Common stickleback consume fewer eggs than white stickleback, and F2 hybrids consume significantly more eggs than either of the F0 cross types. Each data point represents a male, with boxes representing interquartile ranges with the median and whiskers representing 1.5 times interquartile ranges. Significance lettering reflects post‐hoc Dunn's tests.

### 3.3 Little evidence for mate choice against F1 hybrid males or nest architecture as an isolating barrier

There was no evidence that F1 hybrid males were less attractive to females: Mating success (the number of trials before successfully mating) did not differ among male cross types when paired with female commons (Kruskal–Wallis *χ* ^2^ = 0.54, df = 2, *p* = 0.760, *n* = 6 per group) or female whites (Kruskal–Wallis *χ* ^2^ = 2.40, df = 2, *p* = 0.302, *n* = 6 per group), suggesting that male F1 hybrids can readily mate with F0 females under no‐choice conditions.

Since divergent nest architecture could act as a prezygotic isolating barrier if female whites and commons exhibit different nest preferences, we investigated if there was any evidence for assortative mating based on nest architecture. Consistent with previous studies (Behrens et al., 2024), nest architecture significantly differed according to male cross type (Figure S2; Kruskal–Wallis *χ* ^2^ = 10.55, df = 3, *p* = 0.014). Whites built high‐scoring nests (tall and loose), commons built low‐scoring nests (compact and sandy) and F1 hybrids built intermediate nests. There was no evidence that the nest score of F1 hybrid males influenced mating success with female commons (*ρ* = −0.14, *p* = 0.726, *n* = 9) or whites (*ρ* = −0.28, *p* = 0.407, *n* = 11).

Nest architecture might also be important as a postzygotic isolating barrier if, for example it is easier for males to disperse offspring from white‐like nests, or to fan embryos in common‐like nests. We found no evidence that F1 hybrid nest score was correlated with rates of dispersal when mated to female whites (*ρ* = −0.48, *p* = 0.135, *n* = 11). However, F1 hybrid nest score was significantly associated with rates of fanning when mated to female commons (*ρ* = −0.85, *p* = 0.004, *n* = 9), with common‐like nests receiving greater rates of fanning.

### 3.4 Whites and commons hybridize in the wild

Despite significant phenotypic divergence, white and common stickleback exhibit low genetic divergence and are likely experiencing ongoing gene flow (Samuk, 2016). If postzygotic behavioural isolation via dysregulated paternal care is acting as a behavioural isolating mechanism, then F1 hybrids should be detectable in the wild and behavioural sterility could provide an explanation for the limited gene flow among populations. Consistent with this line of reasoning, while wild‐caught commons and whites clustered into distinct genetic groups, putative hybrids were detected as genetically intermediate individuals (Figure 6). The presence of these individuals is the first genetic evidence of ongoing, natural hybridization in this system and suggests that common and white stickleback naturally hybridize in the wild at low levels, with 2.5% of randomly sampled individuals classified as putative hybrids across sympatric sites (Figure 6). Hybrid class (e.g. F1, F2, backcross, etc.) was unable to be determined due to low genetic differentiation between commons and whites. Randomly sampled individuals at sites known to contain only common sticklebacks displayed a single genotypic cluster (Figure S3; BC and MJ clusters), suggesting that the clustering and intermediates found at the white/common sympatric sites are not an artefact of sampling.

**FIGURE 6:**
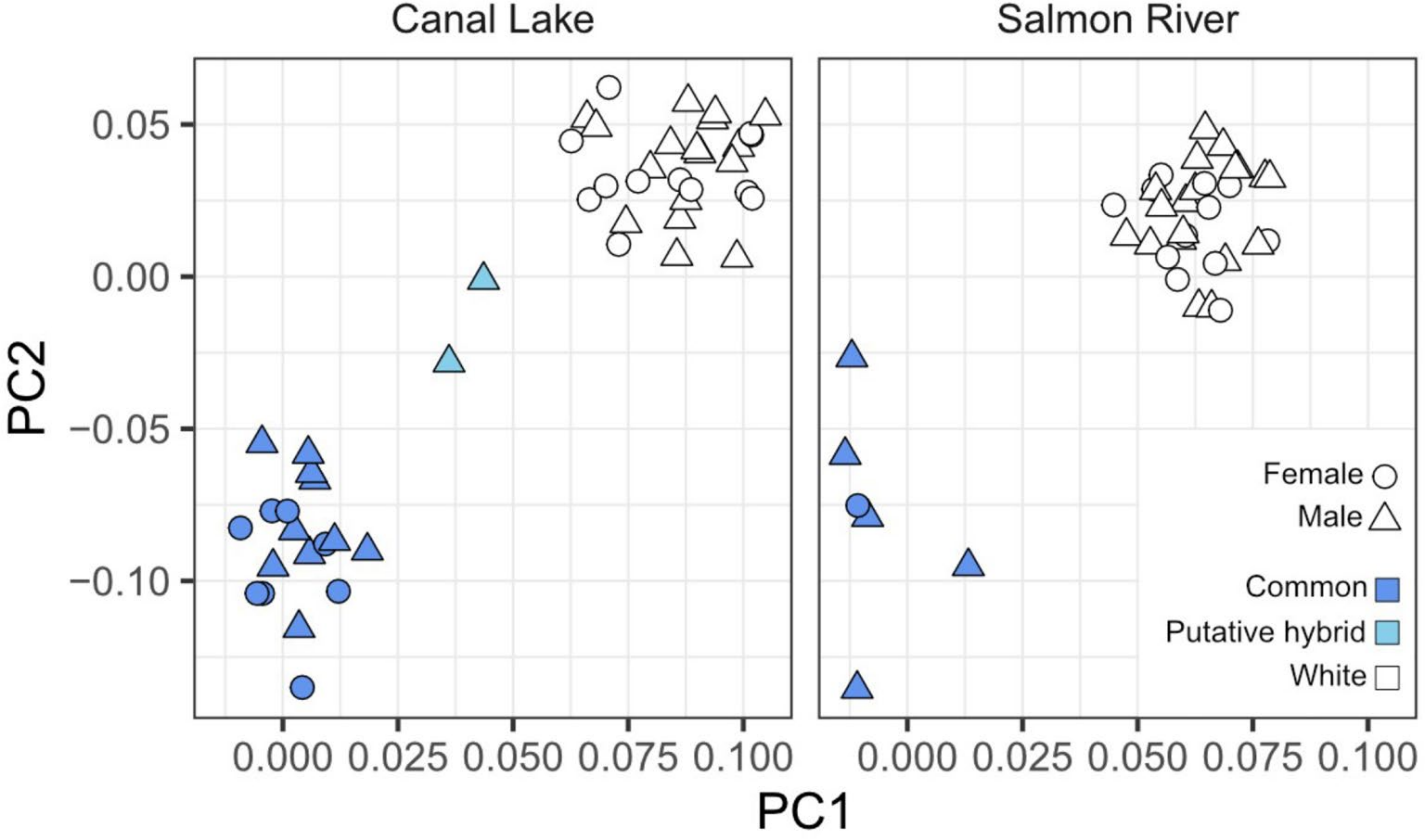
A principal component analysis of RADseq‐derived SNPs from randomly sampled stickleback individuals in two locations where white and common sticklebacks are sympatric (Canal Lake and Salmon River, Nova Scotia). Points are coloured by ecotype (white, common) and sex (male, female) at each site, with putative hybrids indicated. Points are plotted in the same latent space as Figure S3.

We further tested for the presence of hybrid individuals using two additional ancestry analyses on the sympatric and contact‐zone samples (Canal Lake and Salmon River, with Cherry Burton Road as a common‐only reference; 129 individuals total). The first was a supervised Discriminant Analysis of Principal Components (DAPC; Jombart et al., 2010), trained on the 40 WGS samples using their a priori morphological ecotype labels and then used to predict the ecotype of the 89 RAD‐sequenced individuals as held‐out. The second was a sparse non‐negative matrix factorization (sNMF; Frichot et al., 2014), an unsupervised model‐based estimator of individual ancestry proportions that requires no prior labels. Both analyses recovered the same axis of common/white differentiation as the PCA and both identified genetically intermediate individuals concentrated at Salmon River and among the RAD‐sampled fish from Canal Lake (Figures S4 and S5). All individuals identified as putative hybrids by the PCA were also identified as intermediate by both methods, and the direction of admixture (common‐leaning vs. white‐leaning) agreed across analyses. sNMF identified several further admixed individuals that the PCA did not resolve, suggesting that the ~2.5% figure reported above is a conservative lower bound on the rate of hybridization in these sympatric populations.

## 4 DISCUSSION

Speciation is one of the most vexing problems in evolutionary biology, as it is critical for understanding the origins and maintenance of biodiversity but is challenging to study. An essential goal of the field is to identify how traits contribute to speciation, and the study of reproductive isolating mechanisms in the early stages of divergence between populations (before confounding differences have accumulated) has helped to advance our understanding of the speciation process. The radiation of three‐spined stickleback has been an important model in speciation research for these reasons (McKinnon & Rundle, 2002).

This study leverages the white‐common stickleback system to present empirical evidence for a novel behavioural postzygotic reproductive isolating mechanism: behavioural sterility via dysregulated paternal care. The possibility that disrupted parental behaviour might contribute to reproductive isolation was first suggested by Buckley (1966), who found that female hybrid *Agapornis* parrots display altered nest‐building and, if their eggs were fertilized, poor parental care that led to clutch failure. However, these hybrid parrots also suffered almost complete physiological sterility and poor viability (Buckley, 1969), and therefore, their altered parental behaviour likely does little to prevent gene flow. By contrast, in the white‐common stickleback system, there is no evidence for intrinsic postzygotic barriers like hybrid gametic sterility or inviability. Behavioural sterility in the form of dysregulated paternal behaviour in hybrid male stickleback could, therefore, play an important role as a reproductive isolating barrier in this species, and these results add empirical support to theoretical models that have suggested such a pattern (Reyes et al., 2025).

Theory often predicts that isolating mechanisms acting relatively early in reproduction (e.g. timing of breeding, mate choice) are more effective isolating barriers than mechanisms that act later (e.g. hybrid genetic incompatibilities; Mayr, 1963; Coyne & Orr, 2004), but models suggest that partial prezygotic barriers, like assortative mating, can be ineffective without accompanying postzygotic isolation (Irwin, 2020). In the white‐common stickleback system, prezygotic barriers appear to be minimal and/or evidence for them is inconclusive (Behrens et al., 2024; Blouw & Hagen, 1990; Corney & Weir, 2023); therefore, behavioural sterility via dysregulated paternal care may contribute to reproductive isolation in this system, and this (in addition to very recent divergence) could help explain why whites and commons are only weakly genetically differentiated. However, the low rate of hybridization in the wild (~2.5%) suggests that prezygotic barriers, microhabitat preferences and sexual selection against hybrids may be stronger in natural environments, as is observed in other stickleback systems (Bolnick et al., 2015; Vamosi & Schluter, 1999). For example, variation in nest architecture may act as a prezygotic barrier (potential mates prefer specific designs) and as a postzygotic barrier (common‐like nests received greater rates of fanning). Location‐specific factors (e.g. habitat variation, population demographics, demographic history) may also influence hybridization rates, as hybrids were only detected in one of the two sympatric sites, but increased sampling would be required to test this, Additionally, sexually imprinted traits are known to contribute to speciation in stickleback (Kozak et al., 2011), meaning daughters imprinted on a father's traits may affect mate preference and further facilitate speciation.

The specific genetic and behavioural causes of dysregulated paternal behaviour in F1 hybrids are not known, but we suspect that a primary causes of mortality of offspring reared by hybrid males are pathogens, caused by the father's failure to perform an appropriate combination of behaviours to combat fungus and/or filial cannibalism. To prevent fungal infection, male commons tend the nest and remove dead and decaying eggs. By contrast, male whites prevent the spread of pathogens by breaking apart the clutch and scattering the embryos in small clumps. Hybrid fathers performed a mix of behaviours that typify the white and common strategies, and it is possible that these low/combinatorial levels of behaviour may have directly contributed to the death of offspring due to fungal infection. Indeed, dysregulated parental care may have stronger effects in natural conditions, where pathogens and predators are ubiquitous, and field studies could further clarify the strength of postzygotic isolation in this system. However, the mechanistic chain linking dysregulated care to offspring mortality was not fully quantified in this study, and additional studies are required to evaluate this hypothesis.

While the care provided by hybrid fathers may have contributed to the low survival of offspring, we also provide evidence suggesting that white and common reproductive strategies may be incompatible at the genetic level, causing high rates of filial cannibalism in F2 hybrids. Filial cannibalism could also explain why levels of paternal behaviour by F1 hybrid fathers were often low relative to white or common fathers: Embryos elicit caregiving behaviours (Figure 4; see also van Iersel, 1953) and if the F1 hybrid fathers consumed embryos, then there were fewer embryos to fan. Note that although significant cannibalism was observed by F2 hybrids, cannibalism by F1 hybrid fathers was not quantified in this study; clarifying the link between cannibalism rate and dysregulated parental care is a priority for future studies.

The high rates of cannibalism by hybrid fathers could reflect dysregulation of overlapping neural circuits involved in feeding, infant‐directed aggression and parenting. Growing evidence in vertebrates suggests that core conserved neuropeptides, such as galanin, mediate both feeding and care (Fischer & O'Connell, 2017; O'Rourke & Renn, 2015). For example optogenetic stimulation of preoptic galanin neurons was sufficient to switch the behaviour of laboratory male mice from infanticidal to active parental behaviour (Wu et al., 2014). Intriguingly, galanin is also differentially expressed during paternal care (Bukhari et al., 2019), and preoptic galanin neurons are differentially activated during caregiving in commons compared to whites (Maciejewski et al., 2025). Therefore, we hypothesize that genetic incompatibilities may have disrupted parental and feeding circuits, causing hybrid stickleback fathers to consume rather than care for their embryos. However, hybrid filial cannibalism was only quantified across a single F2 hybrid mapping family, and it is possible that genetically based deleterious effects were amplified or diminished in our dataset. Additional studies are required to better understand how neuronal circuits and genomic loci contribute to cannibalism in this system, especially in wild populations.

Consistent with the hypothesis that hybrid males engaged in higher than typical rates of filial cannibalism due to disrupted neural circuits, hybrid male fathers did not preferentially consume unhealthy embryos or unfertilized eggs that were dead or decaying. Instead, many of the embryos found in hybrid males' stomachs were fertilized and developing normally, and filial cannibalism was quantified within 1 h of mating, which is too soon for fungal infections to start or spread. Indeed, under natural circumstances, rates of filial cannibalism by hybrid fathers may be even higher because their embryos fail to develop due to altered care, per above, or because their spiggin—a glue that stickleback fathers use to hold the nest together—might lack antimicrobial properties (Little et al., 2008).

This study focuses on male parental behaviour, but females also likely play a critical role in driving reproductive isolation in this system. Typically, female stickleback produce viscous ovarian fluid that holds eggs together within a clutch. By contrast, the ovarian fluid of female whites is relatively thin (Grant, 1993), which may facilitate the egg scattering behaviour of male whites after fertilization and prevents the offspring from staying ‘all in the same basket’, and thereby vulnerable to total loss by predation or fungus. In support of this hypothesis, our results suggest that the eggs of female whites were easier for males to disperse. These findings prompt future investigation into the adhesiveness of F1 hybrid female clutches (preliminary studies suggest they are intermediate; Bagazinski, A., Maciejewski, M.F., Bell, A.M., personal communication) and raise interesting questions about the outcome of white × common matings and the direction of introgression. For example, if hybridization is limited by hybrid males, but not hybrid females, then females could be driving gene flow between populations. Future studies should examine the reproductive biology of hybrid females (mate choice behaviour, embryo survival, clutch viscosity, etc.) to investigate this possibility.

More than 150 years after Darwin's pivotal discoveries, speciation is still challenging to understand. Identifying the processes involved in speciation is difficult, in part because it requires careful examination of natural history to understand the biology of the study system, and because speciation research frequently requires a retrospective view of events that occurred thousands or millions of years in the past. Identifying reproductive isolating mechanisms and quantifying their effects in systems is therefore key to understanding the speciation process. Our results contribute to this field by suggesting that dysregulated parental care can act as an underappreciated reproductive isolating barrier in stickleback and other animals.

## AUTHOR CONTRIBUTIONS

Colby Behrens and Alison M. Bell conceptualized the project. Alexandra Sumarli, Gina Lucas and Kieran Samuk performed field collections in 2023 and reared organisms. Gina Lucas and Kieran Samuk performed field collections (RADseq) in 2022. Colby Behrens collected behavioural data. Colby Behrens and Meghan F. Maciejewski collected cannibalism data. Gina Lucas performed DNA extractions and normalizations. Gina Lucas and German Lagunas‐Robles performed RADseq library preps. Alexandra Sumarli and Kieran Samuk processed the sequencing data for population genetic analyses. Colby Behrens and Kieran Samuk performed analyses and data visualization. Colby Behrens and Alison M. Bell wrote the original draft. Colby Behrens, Kieran Samuk and Alison M. Bell reviewed and edited the manuscript.

## CONFLICT OF INTEREST STATEMENT

The authors declare no conflicts of interest.

## ETHICS STATEMENT

Experimental protocols were approved by the University of Illinois Urbana‐Champaign IACUC (Protocol #18080 and #21031). Fish were collected from Nova Scotia, CA under the Department of Fisheries and Oceans Maritime permit #366232.

## Supporting information


**Figure S1.** Clutch weights from F0 females. Common and white stickleback did not differ in clutch mass, though artificially‐fertilized clutches were smaller than father‐reared clutches (*t*_60.6_ = −4.90, *p* < 0.001). Each data point represents the clutch from an individual female.
**Figure S2.** Differences in nest architecture. The nest architecture score is a scale (ranging 4–20) incorporating the nest height, nest location, sand content and nest opening. Higher scores represent tall nests with horizontal openings and no sand. Low scores represent compact nests with vertical openings and large amounts of sand. Each data point represents a nest from a single male. Nests from commons and whites significantly differ in overall architecture, while F1 hybrids are intermediate. Significance lettering reflects Tukey HSD post‐hoc tests.
**Figure S3.** Principal component analysis of the jointly filtered SNPs from the randomly sample RADseq individuals (‘RAD’) and WGS samples with known sex and species identities (‘WGS’). Points are coloured by population/sequencing strategy, with shape indicating sex. The separate clustering of BC and MJ is due to known geographic structure in the common stickleback population. BC and MJ are both located in the Bras d'Or Lake, an inland sea on Cape Breton Island, whereas CB/CL/SR are found on the mainland of Nova Scotia.
**Figure S4.** Discriminant Analysis of Principal Components (DAPC) of the 129 mainland samples (Cherry Burton Road common WGS [CB, *n* = 20], Canal Lake white WGS [CL_WGS, *n* = 20], Canal Lake RAD [CL_RAD, *n* = 50], Salmon River RAD [SR, *n* = 39]). The DAPC was trained on the 40 WGS samples (CB and CL_WGS) using their a priori morphological ecotype labels; the 89 RAD‐sequenced individuals (CL_RAD, SR) were then assigned as held‐out via predict.dapc(). (A) DAPC discriminant score (LD1) plotted against the first principal component of the same genotype matrix. Point shape gives population, and point fill gives the posterior probability of assignment to the white ecotype, P(white). The two WGS training populations sit at opposite ends of LD1, and several held‐out RAD individuals from CL_RAD and SR have intermediate LD1 scores. (B) Per‐individual posterior probability of assignment to the common (blue) or white (unfilled) ecotype, sorted within population by P (common). Intermediate posteriors (mixed shading) are present in CL_RAD and SR but absent from the training populations CB and CL_WGS.
**Figure S5.** Unsupervised ancestry estimates from sparse non‐negative matrix factorization (sNMF; LEA::snmf) for the 129 mainland samples (Cherry Burton Road common WGS [CB, *n* = 20], Canal Lake white WGS [CL_WGS, *n* = 20], Canal Lake RAD [CL_RAD, *n* = 50], Salmon River RAD [SR, *n* = 39]). Rows are ancestry proportions for the best run (lowest cross‐entropy across 100 replicates) at *K* = 1 to *K* = 6; columns are populations. Each vertical bar is one individual, sorted within population by ancestry proportion at the corresponding *K*. Cross‐entropy selects *K* = 1, consistent with weak genetic differentiation between the two ecotypes. At *K* = 2, the model still recovers a clear common/white axis: CB sits almost entirely in one component (median *q* = 0.97) and CL_WGS in the other (median *q* = 0.06), while CL_RAD and SR contain many individuals with intermediate ancestry. These intermediates are the same individuals flagged as putative hybrids by the main‐text PCA (Figure 6) and by the DAPC (Figure S4).
**Method S1.** Animal husbandry. Fry were reared in 5‐gallon aquariums and later moved to 20‐gallon aquariums at the University of California Riverside (University of California Riverside IACUC #83). Fish were maintained at 17°C with a 12:12 (L:D) photoperiod and fed brine shrimp, blood worms and mysis shrimp daily. In the spring of 2024, fish were transported to facilities at the University of Illinois Urbana‐Champaign. Fish were maintained at summer conditions (20°C with 16:8 (L:D) photoperiod) and fed a mixture of blood worms, mysis shrimp, brine shrimp and cyclops daily. Experimental protocols were approved by the University of Illinois Urbana‐Champaign IACUC Protocol #24049.
**Method S2.** Sample collection. Stickleback were trapped using unbaited ½” mesh Gee Minnow traps. Twelve minnow traps were set at each site for 8–12 h, after which their contents were placed into aerated buckets containing fresh sea water. All non‐three‐spined individuals were removed from the buckets and returned to the trapping sites. Fifty individual three‐spined sticklebacks were then randomly removed from the buckets and fin clips of approximately 0.5 cm squared were taken using dissecting scissors. The fin clips were immediately placed in 1.5 mL microcentrifuge tubes containing 95% ethanol. All fish were then returned to the trapping sites.
Method S3: Variant Calling and Filtering RADseq and WGS reads were processed independently but followed similar bioinformatics steps unless otherwise stated. Raw RADseq reads were demultiplexed using the process_radtags module in Stacks v 2.6 (Catchen et al., 2013). For both RADseq and WGS datasets, sequence reads were aligned to the three‐spined stickleback reference genome v5 (Nath et al., 2021) using bwa mem (Li & Durbin, 2009). Duplicate reads in the WGS data were removed using Picard (2019). Variant calling was performed using GATK HaplotypeCaller (McKenna et al., 2010). All prior steps were carried out using the snakemake pipeline, grenepipe (Czech & Exposito‐Alonso, 2022). The resulting GVCFs were then merged using the GenomicsDBImport argument in GATK. This was done for each chromosome prior to joint genotyping using GenotypeGVCFs. The resulting vcfs were then subset and filtered in vcftools v 0.1.16 (Danecek et al., 2011). In general, we retained variants that were biallelic snps and followed the GATK best practices hard filters. The separate RADseq and WGS vcfs were then intersected to identify regions shared between both datasets using bedtools v 2.31.0 intersect (Quinlan & Hall, 2010). The intersected VCFs containing shared sites were then merged together using bcftools v 1.16 (Li, 2011). Finally, we filtered the intersected VCF using snpfiltR (DeRaad, 2023) with the following parameters: retaining biallelic SNPs, minimum genotype depth of 5 and quality of 20, maximum missing data 0.85 (i.e. 15% missing data allowed), maximum per sample depth of 40, minimum minor allele count of 1. After intersection and filtering, 4507 high‐quality SNPs were retained. All variant calling and filtering for SNPs were done on the computer cluster provided by the University of California, Riverside High‐Performance Computing Center.

**Table S1.** Accession numbers for sequencing data from all WGS and RAD samples.

**Video S1.** A male common stickleback exhibits typical parental care behaviours, including nest attendance, nest fanning and nest poking. The nest is on the left side of the sandbox.

**Video S2.** A male white stickleback exhibits dispersal behaviour. Shortly after fertilizing a clutch, the male removes embryos from his nest and disperses them into the surrounding environment.

**Video S3.** A male F1 hybrid disperses a clutch from a common female. The clutch is sticky and difficult to separate, so the majority of the clutch is removed in a single dispersal. The male then returns to fan his primarily empty nest.

## Data Availability

Raw RAD sequence data are deposited in the NCBI Sequence Read Archive under BioProject: PRJNA1244444. Raw WGS sequence data are deposited under BioProject: PRJNA1244922. Accession numbers for each sample are listed in Table S1. Behavioural data and code are available from the Dryad Digital Repository DOI https://doi.org/10.5061/dryad.p2ngf1w30 (Behrens, Maciejewski, et al., 2026b). Code for genomic analyses can be accessed at https://github.com/samuk‐lab/ws‐radseq.
